# Insight into development of job-related well-being: the role of four job crafting strategies and psychological needs

**DOI:** 10.3389/fpsyg.2025.1487043

**Published:** 2025-06-26

**Authors:** Lukasz Baka, Monika Prusik, Romuald Derbis

**Affiliations:** ^1^Institute of Psychology, The Maria Grzegorzewska University, Warsaw, Poland; ^2^Department of Psychology, University of Warsaw, Warsaw, Poland; ^3^Institute of Psychology, Opole University, Opole, Poland

**Keywords:** work engagement, exhaustion, job crafting, psychological needs satisfaction, psychological needs frustration, longitudinal study

## Abstract

By applying the job demand-resources and self-determination theories, this three-wave study (with eight-month intervals) examined direct and indirect (via satisfaction and frustration of psychological needs at work) effects of four job crafting strategies on two dimensions of job-related well-being (engagement and exhaustion). It was hypothesized that approach-oriented job crafting related to increasing structural/social job resources and challenge demands leads to higher needs satisfaction, which result in higher engagement, over time. In contrast, avoidance-oriented job crafting related to decreasing hindrance demands is associated with higher needs frustration and further with higher exhaustion. Data were collected among 839 social service workers. All three waves of the study were conducted during the coronavirus pandemic. Structural equation modeling supports these hypotheses in part. The positive direct relation between the crafting of hindrance demands and exhaustion was not confirmed, in contrast to the indirect effect of psychological needs frustration. Of the three approach-oriented job crafting strategies, only increasing structural resources was direct related to work engagement. Increasing structural resources and challenge demands were positively associated with needs satisfaction. Contrary to them increasing social resources resulted in lower needs satisfaction. Higher needs satisfaction led to higher work engagement. The results were discussed in relation to competing motives for building relationships and strengthening one’s own competences.

## Introduction

Over the past two decades, job crafting has become a prominent topic of investigation in work psychology (Frederick and VanderWeele, 2020; Lichtenthaler and Fischbach, 2019). This growing interest is likely linked to the increasing awareness among organizational leaders that employees are not merely passive recipients of workplace conditions but active agents who shape their own work environments (Wrzesniewski and Dutton, 2001). Job crafting (JC) is defined as a bottom-up, proactive behavior initiated by employees (Wrzesniewski and Dutton, 2001), involving the intentional modification of specific job characteristics—such as job demands and job resources—in order to better align work with their individual strengths, values, and needs (Tims et al., 2013). Although job crafting was initially conceptualized as a means to enhance job-related well-being—e.g., by increasing work engagement and reducing burnout (Tims et al., 2013)—subsequent research has revealed that the effects of job crafting depend on the type of behaviors involved (Petrou et al., 2018; Tims et al., 2015; Demerouti et al., 2015; Petrou et al., 2015). Drawing on two dominant theoretical models—the original conceptualization by Wrzesniewski and Dutton (2001) and the Job Demands–Resources (JD-R) perspectives proposed by Tims et al. (2013), and Tims and Bakker (2010)—Zhang and Parker (2019) identified two broad categories of job crafting strategies: approach-oriented strategies (e.g., seeking additional resources or challenges at work) and avoidance-oriented strategies (e.g., reducing or avoiding problematic demands). Meta-analytic findings consistently indicate that the former type—approach-oriented job crafting—is positively associated with well-being at work, including higher levels of work engagement (Frederick and VanderWeele, 2020; Lichtenthaler and Fischbach, 2019). In line with JD-R theory work engagement (WE) is understood as a positive work-related affective-cognitive state of mind, that is characterized by three interrelated components (Schaufeli et al., 2002)—vigor (i.e., high level of energy while working), dedication (i.e., feelings of enthusiasm, significance, a sense of pride and inspiration) and absorption (i.e., intense concentration on work-related activities). The avoidance-oriented strategy, however, appears to be more problematic. Although it was initially suggested that avoiding hindrance demands may serve as a short-term recovery mechanism and a way to enhance job-related well-being (Tims and Bakker, 2010), a growing body of evidence indicates that such behaviors are associated with increased levels of job burnout over time (Tims et al., 2013; Petrou et al., 2018; Tims et al., 2015; Demerouti et al., 2015; Petrou et al., 2015). Job burnout is conceptualized as a long-term consequence of chronic work-related stress, typically resulting from excessive job demands (Bakker and Demerouti, 2017; Bakker et al., 2023). Among the various components of burnout, exhaustion is widely recognized as its core dimension (Maslach et al., 2001), and for this reason, it has been included as a key outcome in the present study. Exhaustion (EXH) refers to a state of extreme physical, emotional, and cognitive depletion, characterized by fatigue, a sense of weariness, and reduced energy levels (Maslach et al., 2001).

While the direct effects of JC on WE and EXH are well documented (Tims et al., 2013; Petrou et al., 2018; Petrou et al., 2015), the psychological mechanisms underlying these effects remain insufficiently understood (Bakker and Demerouti, 2017). Wrzesniewski and Dutton (2001) proposed that employees engage in job crafting primarily to maintain a sense of control, present a positive self-image, and foster constructive relationships with colleagues. These motivations closely align with the three basic psychological needs outlined in Self-Determination Theory (SDT) (Deci and Ryan, 2000): autonomy, competence, and relatedness. Consequently, these needs are considered potential mediators in the relationship JC and job-related well-being. In this three-wave longitudinal study, we examined two distinct constructs—basic psychological needs satisfaction (BPNS) and basic psychological needs frustration (BPNF)—as mediators of the relationship between JC and two dimensions of job-related well-being (WE and EXH). Specifically, adopting a time-lagged design, we tested four hypotheses: (1) the direct effect of approach-oriented job crafting on WE; (2) the direct effect of avoidance-oriented job crafting on EXH; (3) the indirect effect of BPNS in the relationship between approach-oriented job crafting and WE; and (4) the indirect effect of BPNF in the relationship between avoidance-oriented job crafting and EXH.

This study makes a meaningful theoretical contribution by integrating SDT and JD-R framework to better understanding of the mechanisms through which different forms of crafting behaviors influence employee well-being. While previous studies suggest that approach-oriented job crafting is typically associated with positive outcomes (e.g., increased work engagement) and avoidance-oriented job crafting with negative outcomes (e.g., higher levels of job burnout), the specific psychological processes underlying these associations remain underexplored. To address this gap, the present study examines the mediating role of basic psychological needs—both their satisfaction and frustration—as conceptualized within the SDT framework (Deci and Ryan, 2000). More specifically, we propose that approach-oriented job crafting enhances work engagement by fulfilling employees’ basic psychological needs for autonomy, competence, and relatedness, whereas avoidance-oriented job crafting may lead to increased exhaustion by frustrating these same needs. By examining both the “bright” and “dark” motivational pathways, this study moves beyond a simplistic classification of job crafting behaviors as uniformly beneficial or harmful. Instead, it focuses on the underlying motivational quality of these behaviors and their downstream effects on psychological functioning at work. In doing so, the research offers a more nuanced theoretical perspective on how different job crafting strategies are differentially associated with well-being outcomes, depending on the extent to which they facilitate or hinder the satisfaction of core psychological needs.

Importantly, the study employs a three-wave longitudinal design with time lags between measurements of predictors (JC), mediators (BPNS and BPNF), and outcomes (WE and EXH). This methodological approach provides a more robust test of temporal precedence and helps to clarify the directionality of the proposed relationships. Rather than relying on cross-sectional data, which precludes strong inferences about causality, the use of time-lagged data allows us to trace how job crafting behaviors initiated at one point in time lead to subsequent changes in motivational states and, ultimately, to changes in job-related well-being. In doing so, the study strengthens the empirical foundation for theoretical integration between SDT and JD-R, positioning psychological need dynamics as a central explanatory mechanism through which proactive work behaviors shape both positive and negative indicators of employee functioning.

Taken together, this research contributes to a more comprehensive understanding of the effect of job crafting on employees well-being by demonstrating that this process is neither immediate nor uniform, but rather unfold over time through motivational mechanisms grounded in psychological need regulation. These findings have important implications for the refinement of existing theoretical models of job crafting, as well as for the design of workplace interventions that aim to foster sustainable forms of employee proactivity while mitigating potential adverse outcomes.

### General assumptions of JD-R theory

According to the JD-R theory (Bakker and Demerouti, 2017; Bakker et al., 2023), each work has specific job characteristics that can be classified into two general categories called demands and resources. Job demands refer to physical, psychological, social or organizational aspects of a job that require sustained physical and/or psychological effort and are, therefore, associated with related physical and/or psychological costs (Bakker and Demerouti, 2017). Cavanaugh et al. (2000) elaborated this definition by differentiating between challenge and hindrance demands. Challenge demands are perceived by the employee as creating opportunities for personal development—gaining new skills, experiences, broadening horizons, reinforcing a sense of self-efficacy. Hence, they can be a source of positive emotions and result in high well-being (Podsakoff et al., 2007). Conversely, hindrance demands are viewed as barriers that conflict with other duties and hinder the achievement of goals and personal development, therefore they lead to undesirable outcomes, e.g., burnout (Crawford et al., 2010). Job resources, by contrast, refer to these aspects of the job that may reduce job demands, are functional in achieving work goals, and stimulate personal growth, learning and development (Schaufeli et al., 2002). Job resources play the crucial role in development of work engagement (Cavanaugh et al., 2000).

### Job crafting from JD-R theory perspective

The concept of job crafting was originally proposed by Wrzesniewski and Dutton (2001) and subsequently elaborated within the Job Demands–Resources (JD-R) framework (Bakker and Demerouti, 2017; Bakker et al., 2023). In line with JD-R theory, JC refers to a set of proactive behaviors through which employees attempt to balance job demands and resources with their personal values, abilities, and needs. Tims and Bakker (2010) identified four categories of crafting behaviors that employees may engage in to achieve this balance: increasing structural job resources (e.g., enhancing autonomy, skill variety, and opportunities for development); increasing social job resources (e.g., seeking support and performance feedback); increasing challenge demands (i.e., seeking tasks that foster personal growth and the attainment of professional goals); and decreasing hindrance demands (i.e., minimizing or avoiding aspects of the job that obstruct development and goal attainment). The first three strategies are classified as approach-oriented crafting (Zhang and Parker, 2019), reflecting active, constructive efforts to expand one’s personal and job-related resources—both structural (e.g., autonomy) and social (e.g., support)—as well as to initiate new challenges that build self-efficacy and adaptive coping skills (Tims and Bakker, 2010). In contrast, decreasing hindrance demands represents an avoidance-oriented strategy, which is qualitatively distinct from the other three (Zhang and Parker, 2019). This form of JC involves efforts to avoid or delay tasks perceived as overly taxing, emotionally draining, or detrimental to professional functioning and psychological well-being (Tims and Bakker, 2010).

By proactively adjusting levels of resources and challenges to their personal preferences, employees can reshape their work to be more stimulating, rewarding and engaging for them (Tims et al., 2013; Tims and Bakker, 2010). Indeed, the positive link between approach-oriented crafting and engagement has been confirmed in numerous studies (Frederick and VanderWeele, 2020; Lichtenthaler and Fischbach, 2019). Although in the light of JD-R theory (Bakker and Demerouti, 2017; Bakker et al., 2023), reducing hindrance job demands should result in higher well-being at work (Tims and Bakker, 2010), several studies do not confirm this regularity (Demerouti et al., 2015; Petrou et al., 2015). For example, it was demonstrated that the more employees reduce their hindrance demands, the less they are engaged in work (Petrou et al., 2018; Tims et al., 2015) and the more they feel burned out (Demerouti et al., 2015; Petrou et al., 2015). Positive link between decreasing of hindrances and job burned was also confirmed in a meta-analysis (Tims et al., 2013). Perhaps, the effort to reduce hindrance demands is energy-consuming in the long run (Demerouti et al., 2015). It is also possible that avoiding unpleasant tasks or postponing them in time makes employees think about them even more and “chew” them both in and outside of work (Cropley and Zijlstra, 2011), which in the long run results in draining resources and increasing exhaustion (Toyama et al., 2022). Based on the presented theoretical premises and the results of the cited studies, the first two hypotheses expect that:

*H1*: Crafting job resources and challenge demands is positively related to work engagement, over time.

*H2*: Crafting hindrance demands is positively related to exhaustion, over time.

### SDT and job-related well-being

SDT is a meta-theory of human motivation that posits an innate tendency in individuals to act in self-directed and intrinsically motivated ways (Deci and Ryan, 2000; Ryan and Deci, 2000). According to SDT, people are naturally inclined to seek coherence with their environment and integration into broader social contexts, aiming to self-actualize and apply their inherent talents and predispositions in socially meaningful ways (Ryan and Deci, 2002). Full self-actualization and integration, however, are possible only when the social environment supports the satisfaction of three fundamental psychological needs: autonomy, competence, and relatedness (Deci and Ryan, 2000). Autonomy refers to the experience of volition and the belief that one can influence one’s own actions and immediate environment. Competence involves the perception of effectiveness in dealing with challenges, achieving goals, and mastering difficult tasks. Relatedness encompasses the capacity to establish and maintain close, meaningful relationships, along with a sense of mutual trust and respect (Deci and Ryan, 2000).

Within SDT, basic psychological needs are conceptualized as “nutriments that must be procured by a living entity to maintain its growth, integrity, and health” (Baard et al., 2004). Just as plants require water, minerals, and sunlight to thrive, SDT posits that human flourishing depends on the satisfaction of these basic psychological needs. When these needs are met, individuals are more likely to actualize their potential, function optimally, and experience psychological well-being. Conversely, the frustration of these needs is associated with maladaptive functioning and various forms of ill-being (Vansteenkiste and Ryan, 2013). Therefore, basic psychological needs satisfaction (BPNS) and basic psychological needs frustration (BPNF) are treated as two distinct but interrelated constructs (Warburton et al., 2020). Previous research has consistently demonstrated the relevance of both constructs for understanding job-related well-being, including their associations with higher levels of work engagement (Toyama et al., 2022; Van den Broeck et al., 2008) and lower levels of burnout (Van den Broeck et al., 2016; Vander Elst et al., 2012).

### Job crafting and psychological needs

Psychological needs are context-responsive constructs (Vansteenkiste and Ryan, 2013), therefore their satisfaction or frustration depend on the environment in which employees function (Vansteenkiste and Ryan, 2013; Coxen et al., 2021) as well as on their individual involvement in shaping work conditions, including crafting behaviors (Toyama et al., 2022; Slemp and Vella-Brodrick, 2014). In the work context, autonomy satisfaction occurs when the worker experiences psychological freedom, choice and creation, while autonomy frustration is the feeling of being controlled and under pressure. Competence satisfaction includes feeling effective in carrying out job tasks, coping with challenges, achieving high professional goals and being appreciated by others, while the frustration of this need leads to a feeling of inadequacy and failure in the performance of tasks for which an employee is responsible. Relatedness satisfaction involves the sense of being warmly connected to people from one’s work environment (e.g., co-workers, clients), whereas relatedness frustration denotes feelings of loneliness, ostracism or rejection (Ryan and Deci, 2017; Williams, 2009).

By job crafting related to increasing job resources and challenges and adapting them to individual preferences, employees can increase the fit between job demands and their own skills and abilities as well as between rewards/supplies provided by the work environment and their individual needs (Edwards and Van Harrison, 1993). In this way, employees create a more resourceful workplace (Hobfoll et al., 2018) and also satisfy their BPN (Toyama et al., 2022; Slemp and Vella-Brodrick, 2014). Indeed, several studies have shown that the BPNS is linked with access to job resources (Van den Broeck et al., 2016; Coxen et al., 2021; Baka et al., 2022) and challenging job demands (Albrecht, 2015; Olafsen and Frølund, 2018). The structural and social resources enable employees to maintain control over work (autonomy need), improve professional skills and achieve set goals (competence need), as well as exchange support with other colleagues (relatedness need). Challenge demands, in turn, can lead to the acceleration of one’s professional career, increase in salary, and consequently higher independence at work (autonomy need), the strengthening of one’s sense of self-efficacy and the acquisition of new skills (competence need), as well as the expansion of one’s social network (relatedness need).

When employees have the three basic needs satisfied, they function “better” in workplace (Trépanier et al., 2015; Van Hooff and De Pater, 2019) and experience higher levels of job-related well-being (Slemp and Vella-Brodrick, 2014; Gillet et al., 2012), including higher engagement (Toyama et al., 2022; Van den Broeck et al., 2008). Thus, it can be expected that psychological needs will mediate the longitudinal relationship between crafting behaviors and engagement, in such a way that higher JC will lead to greater BPNS, which in turn will result in higher WE, over time. Although such an indirect effect has been tested (Toyama et al., 2022; Slemp and Vella-Brodrick, 2014), the previous studies were conducted in the cross-sectional research paradigm, with all its limitations (Maxwell et al., 2011).

*H3*: Psychological needs satisfaction mediates the longitudinal effect of job crafting related to increasing job resources and challenge demands on work engagement.

Excessive job demands frustrate employees’ psychological needs (Vander Elst et al., 2012; Trépanier et al., 2015), hence, as some authors postulated (Tims et al., 2013; Petrou et al., 2018), lowering these demands should result in a higher needs satisfaction and a better person-environment fit, in the context of balance between demands and abilities (Edwards and Van Harrison, 1993). However, several studies do not confirm this regularity (Toyama et al., 2022; Travaglianti et al., 2016). Conversely, they rather indicate that taking activities related to reducing job tasks perceived as hindrance may be the source of the BPNF. Avoiding difficult situations and stressful interactions in workplace or postponing unpleasant job tasks will not help the employee solve the problems that are the source of individual constraints and tension. The difficulties related to them do not disappear. Instead, the demands accumulates and the employee is constantly worried about delayed tasks, which drains their energy and can be a source of frustration. Indeed, a recent study conducted by Toyama et al. (2022) confirmed that reduction of hindrance demands intensifies needs frustration. The authors argue that by avoiding difficult and demanding job tasks, employees weaken their control over their work (autonomy frustration) and miss the opportunity to acquire new knowledge and skills, which lowers their sense of self-efficacy (competence frustration). It is also possible that the implementation of such passive strategies unexpectedly burdens other employees with responsibilities (Tims et al., 2015), which may worsen interpersonal relations or prompt social rejection (relatedness frustration). Additionally, some studies showed that BPNF may lead to deterioration of job-related well-being (Van den Broeck et al., 2016; Vander Elst et al., 2012), including burnout (Toyama et al., 2022; Travaglianti et al., 2016).

Based on the cited studies, it is expected that needs frustration will mediate the longitudinal relation between reduction of hindrance demands and exhaustion, such that higher decreasing hindrance demands frustrate BPN more strongly, and this in turn leads to higher EXH, over time. To the best of our knowledge, the indirect effect of BPNF in this research context has not been tested in any prospective study.

*H4*: Psychological needs frustration mediates the longitudinal effect of job crafting related to decreasing hindrance demands on exhaustion.

### Social service professionals

Our research was conducted among social service professionals—individuals who work in direct contact with people receiving care, support, education, or counseling services (e.g., social workers, healthcare personnel, educators, and other frontline staff). Social service workers represent an occupational group at elevated risk for job strain, as their daily responsibilities often involve engaging with individuals facing diverse social, health, and educational challenges. This work entails heightened emotional demands, potential interpersonal conflicts, and the need to respond to crisis situations (Giménez-Bertomeu et al., 2024; Sá and Azevedo, 2020). The necessity of forming close, emotionally involved relationships with clients may, over time, contribute to diminished psychological well-being among these workers—manifesting in increased job burnout and decreased work engagement, as evidenced by both turnover intentions and actual turnover rates (Kim and Stoner, 2008; Mercado et al., 2022; Ravalier et al., 2021).

Many existing reviews on the prevalence of job burnout syndrome have primarily focused on healthcare professionals. Although these studies vary in their reported prevalence rates, they consistently indicate that a substantial proportion of workers are affected. For example, Woo et al. (2020), in a study involving an international sample of nurses, found that more than 11% exhibited symptoms of job burnout. Regarding exhaustion—often considered the core component of burnout—prevalence rates have been reported at 33% among primary care physicians (Shen et al., 2022), 27% among mental health professionals (O’Connor et al., 2018), and nearly 44% among healthcare workers in general (Ghahramani et al., 2021). Similar trends have been observed among other social service professionals. For instance, 28% of social workers were found to experience mental health problems (Straussner et al., 2018), and 30% had been diagnosed with job burnout (Abu-Bader, 2000). A more recent meta-analysis of 24 studies conducted between 1990 and 2023, encompassing 16,962 social service workers across seven countries, estimated that 20% experience full-syndrome burnout, while 50% report symptoms of exhaustion (Giménez-Bertomeu et al., 2024). In comparison, a meta-analysis of nine studies from eight countries on teacher burnout reported prevalence rates as high as 52% within that professional group (Ozamiz-Etxebarria et al., 2023).

Also, reduced work engagement and associated staff turnover is a major issue in social service workers (Kim and Stoner, 2008; Mercado et al., 2022; Ravalier et al., 2021). High turnover in social care organizations leads to a number of operational challenges, including rising costs and declining quality of care, which ultimately affects service delivery (Hom et al., 2017; Pharris et al., 2022). Furthermore, turnover negatively affects both professionals and clients contributing to lower ratings of service quality, reduced client trust and increased anxiety among professionals (De Croon et al., 2004). This highlights the need to understand the mechanism for developing work engagement among social service workers.

## Materials and methods

### Participants

The sample study includes 839 Polish human service professionals, belonging to three occupational sectors: health care (*n* = 282), education (*n* = 264) and customer service (*n* = 292). The study was conducted in three waves, with eight-month intervals between the measurements, at the institutions and organizations, where the respondents were employed. The first wave of the study was carried out between June and September 2020, while the second and third waves after eight and 16 months, respectively. Each participant was treated in accordance with the ethical guidelines of the Helsinki Declaration. They received a hard copy of the questionnaires together with a letter explaining the aim of the study. Each of them gave written informed consent to participate in the research. Confidentiality of data and anonymity were provided. Participants were asked to reply to questionnaires and then to seal the questionnaires in envelopes which were then collected by the interviewers. In order to be able to match individual participants in each wave of the study, they received anonymizing identification codes.

The optimal length of time intervals between measurement waves remains a subject of ongoing debate in the literature (Little, 2024). Identifying this “optimal interval” is inherently complex, as it depends on several factors, including the nature of the phenomena under investigation, the specific relationships being tested, and characteristics of the sample (e.g., accessibility and stability of the participant pool). The complexity increases further in mediation models, where the optimal time lag for the predictor (X) to influence the mediator (M) may differ from the time required for the mediator to exert an effect on the outcome (Y) (Little, 2024). For instance, while an eight-month interval may be sufficient for psychological needs to be satisfied as a result of job crafting behaviors, this period may not be adequate for the downstream effects of need satisfaction on work engagement to fully materialize. Given these considerations, our study adopted a pragmatic approach. We assumed that a minimum interval of 6 months would be necessary to capture meaningful psychological changes, while keeping the maximum interval below 1 year to reduce participant attrition and ensure sample retention over time.

Out of 2,000 distributed questionnaires, 1,315 were completed in the first step of the study (T1), 1,025 (78% of the original pool) in the second (T2) and 839 (64% of the original pool) in the final stage. The analyzed group consisted of 601 women (71.6%) and 238 men (28.2%), χ^2^(1) = 158.11, *p* < 0.001, between 20 and 71 years of age (*M* = 43.5084, *SD* = 10.80). Work experience ranged from 1 to 50 years (*M* = 19.30, *SD* = 10.59). A one-way between-subjects ANOVA test showed some significant differences but of medium magnitude (based on effect sizes) in the distribution of age among three occupational groups, *F*(2, 832) = 28.77, *p* < 0.001, η^2^ = 0.07. Health services workers were on average older than education (*p* = 0.001) and customer service employees (*p* < 0.001); also customer service employees were older than education workers (*p* < 0.001). There were also significant, but of low magnitude, differences in the length of service, *F*(2, 821) = 10.58, *p* < 0.001, η^2^ = 0.03, with health services workers having higher seniority in comparison to education (*p* = 0.023) and customer service employees (*p* < 0.001). According to additional attrition analysis using logistic regression basic sociodemographic characteristics (age, gender, seniority at work, occupational group coded as dummies) were not significantly related for drop-out from wave 1 to wave 2, χ2(8) = 11.16, *p* = 0.193, Nagelkerke’s *R* = 0.01, and from wave 2 to wave 3, χ2(8) = 6.54, *p* = 0.587 Nagelkerke’s *R* = 0.01. None specific factor was identified as being a source of drop out.

### Measurements

*Job crafting* was measured with the Job Crafting Scale (JCS) (Tims et al., 2012). This tool assesses four job crafting strategies via 21 items: increasing structural resources (5 items, e.g., *I try to learn new things at work*), increasing social resources (5 items, e.g., *I ask my supervisor to coach me*), increasing challenge job demands (5 items, e.g., *I try to make my work more challenging by examining the underlying relationships between aspects of my job*), and decreasing hindrance job demands (6 items, e.g., *I manage my work so that I try to minimize contact with people whose problems affect me emotionally*). All items were rated on a five-point Likert scale ranging from 1 (never) to 5 (often). Cronbach’s alphas were between 0.77 and 0.81 for increasing structural resources across three measurements occasions (T1–T3), *α* = 0.83–0.87 for increasing social resources, α = 0.83–0.87 for increasing challenge job demands, and α = 0.78–0.84 for decreasing hindrance job demands.

*Basic psychological needs* were assessed by the work-related version of the Basic Psychological Need Satisfaction and Frustration Scale (BPNSFS-Work Domain) (Schultz et al., 2015). The scale consists of 24 items, four for each of the six subscales (i.e., autonomy satisfaction, autonomy frustration, relatedness satisfaction, relatedness frustration, competence satisfaction and competence frustration). The respondents had answered questions concerning their feelings about their jobs during the previous 4 weeks (e.g., *At work, I feel capable at what I do*), on a 7-point response scale ranging from 1 (strongly disagree) to 7 (strongly agree). The aggregated index of satisfaction of needs (α = 0.89–0.90 for T1–T3) and frustration of needs (α = 0.87–0.89 for T1–T3) were used in the present study.

*Exhaustion* was measured using the subscale of the Oldenburg Burnout Inventory (OLBI) (Demerouti et al., 2003), that consists of 8 items. Participants rated items such as *There are days when I feel tired before I arrive at work* on a 4-point scale from 0 (completely agree) to 6 (completely disagree). Cronbach’s alpha was between α = 0.80–0.81.

*Work engagement* was measured using the Utrecht Work Engagement Scale (UWES) (Schaufeli et al., 2002). According to this concept, work engagement includes vigor (6 items, e.g., *At my work, I feel bursting with energy*), dedication (5 items, e.g., *I am enthusiastic about my job*) and absorption (6 items, e.g., *I am immersed in my work*). The answering scale ranged from 0 (never) to 6 (always). For the analyses, we used a composite score of all subscales (Cronbach’s α ranged 0.94–0.95).

### Analytical procedure

The analytical procedure consisted of several consecutive steps. First, we checked whether all necessary assumptions for the planned statistical techniques were met. These involved screening for: normality of constructs, linearity between constructs, number and type of missing data, as well occurrences of outlying cases. As no significant departures were found, we calculated the basic descriptive statistics and correlational matrices. The main procedure involved structural equation modeling (SEM) using IBM Amos ver. 28 and ver. 27. SEM is a widely known statistical technique which allows for, among other things, the simultaneous analysis of a large number of independent but also dependent constructs, instead of testing for individual dependencies in step-by-step procedures (Mercado et al., 2022). The SEM framework also allows for the testing of mediational effects and has been used for this purpose in the presented paper.

## Results

**Figure 1:**
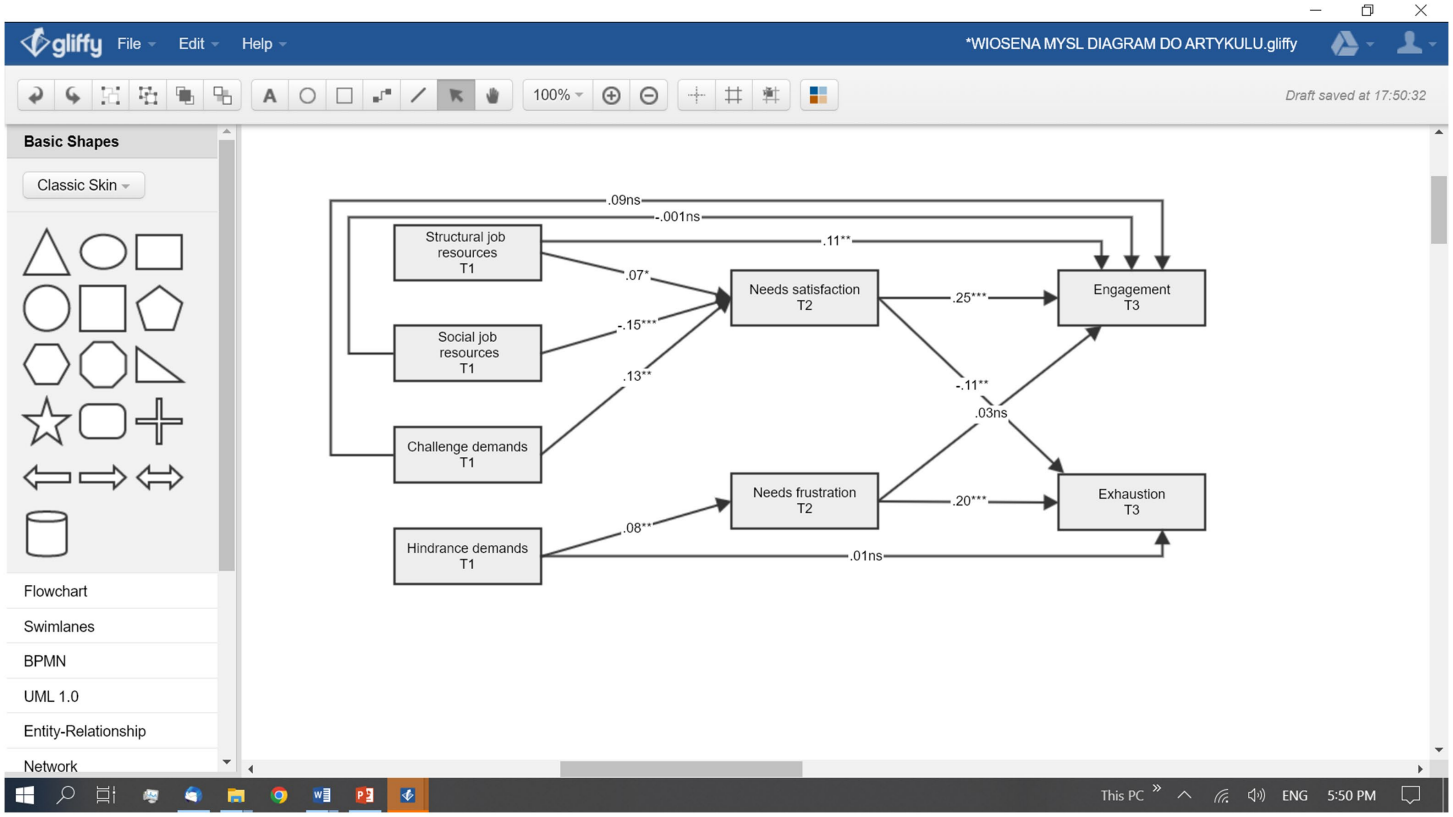
Structural equation model for hypothesized relations between study constructs. Covariates were omitted for clarity. ^*^p < 0.05, ^**^p < 0.01, ^***^p < 0.001.

The basic descriptive statistics for the study constructs together with a zero-order correlational matrix for within time measurement points are included in Table 1. The correlation matrix for the study constructs across time points is presented in Appendix Table 1A. In order to test our hypotheses, we undertook a direct approach and created a model representing only hypothesized paths (Figure 1). This type of approach could be described as a strictly confirmatory approach to SEM, in which the purpose of modeling is to check whether the hypothesized model fits the data or not (Collier, 2020). Goodness of fit indices for the investigated model are presented in Table 2, while the results for the hypothesized model are presented in Figure 1. The model had an overall acceptable fit even though the ratio of χ^2^/df was relatively high but, still, its maximum value was acceptable. The criteria used to assess the goodness of fit were as follows: incremental fit index (IFI) > 0.90–0.95, root mean square error of approximation (RMSEA) < 0.05–0.08, *p* of Close Fit (PClose) > 0.05, standardized root mean square residual (SMRM) < 0.06–0.08, and relative chi-square test (χ^2^/df) < 3.0–5.0 (Kline, 2016; Schumacker and Lomax, 2004; Sharma, 1996). Based on specific regression coefficients, it could be stated that our first two hypotheses (H1 and H2) were partly confirmed. JC related to increasing structural job resources was significantly related to higher WE but social job resources and challenge demands were not directly related to WE as hypothesized (Figure 1). This partly confirms H1 but does not fully align with the results of the correlational analysis (Table 1; Appendix Table 1A) according to which all three constructs (structural job resources, social job resources, challenge demands) were positively related to higher WE. H2 was not confirmed, as JC related to decreasing hindrance demands was not significantly related to EXH. The same pattern of results was pre-confirmed by the correlational analysis (Table 1; Appendix Table 1A). In order to test the hypothesized mediational effects, we ran a mediational analysis using the “indirect effect” estimand for IBM Amos ver. 27 (Gaskin et al., 2020). The results for four hypothesized mediational effects are presented in Table 3. H3 was partly confirmed and H4 was entirely confirmed. BPNS mediated the longitudinal effect of JC related to increasing job resources on WE for social job resources and challenge demands but not for structural job resources. The mediational effect of BPNS on the relationship between structural job resources and WE was only marginal in nature (statistical tendency) and, thus, H3 was only partially confirmed. H4 was fully confirmed as BPNF mediated the link between reducing hindrance demands and EXH, as postulated.

**Table 1:** Descriptive statistics and zero-order correlations for study variables within measurement points, *N* = 839.

Construct	*Min*	*Max*	*M*	*SD*	(1)	(2)	(3)	(4)	(5)	(6)	(7)
Time 1											
1. Structural job resources	1.80	5.00	3.93	0.59	--						
2. Social job resources	1.00	5.00	2.99	0.87	0.27^***^	--					
3. Challenge demands	1.00	5.00	3.27	0.74	0.56^***^	0.66^***^	--				
4. Hindrance demands	1.33	5.00	3.43	0.65	0.26^***^	0.44^***^	0.45^***^	--			
5. Needs satisfaction	1.83	7.00	5.22	0.94	0.46^***^	0.01	0.24^***^	0.12^***^	--		
6. Needs frustration	1.00	6.50	3.00	1.09	−0.26^***^	0.18^***^	0.05	0.15^***^	−0.48^***^	--	
7. Engagement	0.53	6.00	4.26	0.91	0.56^***^	0.24^***^	0.46^***^	0.18^***^	0.50^***^	−0.22^***^	--
8. Exhaustion	1.00	3.75	2.19	0.53	−0.32^***^	0.01	−0.18^***^	−0.01	−0.40^***^	0.46^***^	−0.36^***^
Time 2											
1. Structural job resources	1.20	5.00	3.88	0.62	--						
2. Social job resources	1.00	5.00	3.08	0.81	0.34^***^	--					
3. Challenge demands	1.00	5.00	3.32	0.80	0.66^***^	0.61^***^	--				
4. Hindrance demands	1.17	5.00	3.53	0.69	0.33^***^	0.35^***^	0.47^***^	--			
5. Needs satisfaction	2.08	7.00	5.11	0.90	0.46^***^	−0.02	0.20^***^	0.17^***^	--		
6. Needs frustration	1.00	6.00	2.98	0.98	−0.23^***^	0.16^***^	0.09^*^	−0.01	−0.52^***^	--	
7. Engagement	0.00	6.00	4.22	0.94	0.54^***^	0.33^***^	0.45^***^	0.16^***^	0.40^***^	−0.17^***^	--
8. Exhaustion	1.00	3.75	2.20	0.49	−0.34^***^	−0.04	−0.10^**^	−0.04	−0.39^***^	0.40^***^	−0.32^***^
Time 3											
1. Structural job resources	1.80	5.00	3.89	0.61	--						
2. Social job resources	1.00	5.00	3.07	0.88	0.41^**^	--					
3. Challenge demands	1.00	5.00	3.31	0.83	0.61^**^	0.66^***^	--				
4. Hindrance demands	1.00	5.00	3.65	0.68	0.25^**^	0.31^***^	0.37^***^	--			
5. Needs satisfaction	1.67	7.00	5.18	0.90	0.46^**^	0.05	0.25^***^	0.21^***^	--		
6. Needs frustration	1.08	7.00	3.03	1.08	−0.19^**^	0.18^***^	0.11^**^	0.00	−0.52^***^	--	
7. Engagement	0.76	6.00	4.23	0.95	0.52^**^	0.28^***^	0.43^***^	0.23^***^	0.49^***^	−0.23^***^	--
8. Exhaustion	1.00	4.00	2.18	0.51	−0.30^**^	−0.01	−0.13^***^	−0.06	−0.45^***^	0.50^***^	−0.36^***^

M, Mean; SD, standard deviation. *p < 0.05; **p < 0.01; ***p < 0.001.

**Table 2:** Goodness of fit indices for the investigated models, *N* = 839.

Goodness of fit indices	χ^2^	*df*	*P*	χ^2^*/df*	RMSEA 90[LO; HI]	PClose	SRMR	IFI	AIC
Model	37.28	8	<0.001	4.66	0.07 [0.05, 0.09]	0.094	0.04	0.98	93.28

RMSEA, root mean square error of approximation; PClose, P of close fit; SRMR, standardized root mean square residual; IFI, incremental comparative fit index; AIC, Akaike’s information criterion. χ^2^, chi-squared; df, degrees of freedom.

**Table 3:** Significance of indirect effects for hypothesized mediational effects, *N* = 839.

Mediational path	*B*	Low	High	*p*	β
Structural job resources > Needs satisfaction > Engagement	0.03	0.01	0.06	0.054	0.02
Social job resources > Needs satisfaction > Engagement	**−0.04** ^***^	−0.07	−0.02	0.001	**−0.04** ^***^
Challenge demands > Needs satisfaction > Engagement	**0.04** ^**^	0.02	0.08	0.007	**0.03** ^**^
Hindrance demands > Needs frustration > Exhaustion	0.01^**^	0.01	0.02	0.006	0.02^**^

^*^p < 0.05, ^**^p < 0.01, ^***^p < 0.001. Significant coefficients of regression are presented in bold.

Even though the indirect effects were mostly significant and we were able to observe significant mediation effects, not all underlying paths were significant or converged along the predicted directions. The significance of directional paths including total effect are not mandatory for claims regarding significance of mediation (Hayes, 2018; Zhao et al., 2010). According to our analysis, only one total effect was significant (increase of structural job resources on WE). Paths b were all significant in an expected direction (BPNS related to WE, and BPNF related to EXH). Most paths also followed the expected direction (structural job resources and challenge demands positively related to BPNS, and hindrance demands related to higher BPNF) except one problematic path a (social job resources negatively related to BPNS). A negative relationship between increasing social job resources and BPNS is, however, partly backed up by the results of correlational analysis (Appendix Table 1A). In summary, and according to the typology proposed by Straussner et al. (2018), three significant mediational effects might be described as “indirect-only mediations.”

## Discussion

The aim of this three-wave study was to better understanding of the process by which JC leads to higher job-related well-being, measured by WE and EXH. By applying JDR and SDT theories (Bakker and Demerouti, 2017; Deci and Ryan, 2000), the direct effects of four strategies of JC on well-being and the mediational effects of satisfaction and frustration of the three psychological needs were tested. It has been hypothesized that crafting related to the seeking of job resources (structural and social) and challenge demands will result in an increase in WE, directly and indirectly, via high BPNS. Crafting related to the reduction of hindrance demands, in turn, will lead to lower EXH, directly and indirectly via high BPNF.

The results only partially confirmed the expected direct links. Of the three approach-oriented strategies, only the increase of structural resources (but not social resources and challenge demands) was related to high engagement, measured after 16 months. The obtained findings partially confirm the results of previous studies, which show that seeking structural job resources is the strongest positive predictor of work-related well-being outcomes, e.g., engagement and performance (Frederick and VanderWeele, 2020; Lichtenthaler and Fischbach, 2019). This strategy is the most frequently used by both upper-level (Roczniewska and Puchalska-Kamińska, 2017) and lower-level (Oprea et al., 2020) employees, which may suggest that crafting this kind of resources (seeking job control, creating development opportunities) is the easiest strategy that employees can implement on their own (Toyama et al., 2022). This would partly explain the lack of observed relationships among other two approach-oriented crafting behaviors with WE.

The expected negative link between avoidance-oriented crafting behaviors and exhaustion, measured after 16 months, was not confirmed. The findings of the studies conducted so far, however, show that this relationship is particularly unclear. Some studies have found no link between these constructs (Tims et al., 2013), while others have found a positive (Demerouti et al., 2015; Petrou et al., 2015; Demerouti et al., 2017) or negative relationships (Tims and Bakker, 2010; Bakker et al., 2023). The obtained lack of significant dependence can be explained in several ways. The first way refers the specificity of the surveyed professional group. In some occupations, the many arduous job responsibilities that create tension are unavoidable. They are somehow inscribed in daily duties and closely related to the profession (Toyama et al., 2022). For example, among doctors and nurses such job conditions include contact with chronic diseases, death and potentially infectious material, such as blood, secretions, excreta (Baka and Prusik, 2021). Among teachers, these may be aggression on the part of students, work with the least talented students and the demanding attitudes of parents (Skaalvik and Skaalvik, 2007). Over the course of their professional careers, employees learn that trying to avoid these unpleasant job tasks and uncomfortable situations is ineffective in the long term, and they develop other (more proactive) coping strategies. Moreover, avoiding or postponing hindrance-related tasks, job conditions or relations with other people does not solve the problems that underlie these restrictions. The unpleasant tasks, tiring co-workers or rude superiors that a worker tries to avoid will not disappear from working life. Therefore, sometimes more proactive solutions, such as competency training or improving relations with superiors and colleagues, may be more efficacious (Toyama et al., 2022).

The analyses clearly confirmed the mediational effects of needs satisfaction. Three approach-oriented job crafting strategies (related to increase of resources and challenge demands) were associated with needs satisfaction after 8 months, and this, after another 8 months, led to higher WE. A note should be made, however, of the fact that the sign of the relationship between seeking social resources and needs satisfaction turned out to be negative and, therefore, inconsistent with both the results of previous studies (Slemp and Vella-Brodrick, 2014; Bakker and Oerlemans, 2019) and our predictions. This unexpected connection sign may be explained at least two ways. The first of them refers to the threat-to-self-esteem model, which posits that supportive actions contain elements of both support and threat (Deelstra et al., 2003; Gray et al., 2020). On the one hand, employees who use social networks at work (i.e., actively seek help, set up their own network of contacts), build relationships with other members of the organization and solve problems more efficiently, which improves their well-being (Mathieu et al., 2019). On the other hand however, by using the help of others in daily duties, they do not create opportunities for themselves to acquire new knowledge, develop skills and build their own personal resources (Tims et al., 2012; Schultz et al., 2015). Then, support from co-workers or supervisors may be perceived as threatening (Bamberger, 2009), because it undermines sense professional competences of employees (Beehr et al., 2010) and over time leads to lower job control and decrease in motivation to take new challenges at work (Tims et al., 2012; Schultz et al., 2015). The second explanation relates to the negative effects of overusing the support and kindness of other employees. Frequently turning to co-workers for help or support in solving professional problems can make them feel exploited and manipulated by someone who constantly makes requests of them (Tims et al., 2013). Consequently, they will avoid such collaborators or reject their requests, which may be interpreted by them as exclusion and weaken the commitment in daily life activities as well as an inner interest in the affairs of other employees. It is also possible that those employees who seek social support the most are those who have the lowest level of skills and professional qualifications or are simply the laziest; hence, other employees will be reluctant to help them, because in the future they will not be able to count on their reciprocity (Peeters et al., 1995).

The mediational role of BPNF has been also confirmed. As expected, reducing hindrance demands intensified needs frustration (after 8 months), which in turn led to higher EXH, in the same length of time. This observed regularity brings quite an interesting contribution to job crafting concept. Although reducing hindrances was originally theorized as crafting strategy that helps employees to protect their own resources while keeping “good” health (Tims et al., 2013), this beneficial effect seems to be weakly confirmed empirically (Frederick and VanderWeele, 2020; Lichtenthaler and Fischbach, 2019). The results of some studies rather indicate that such a defensive behaviors may be associated with a weakening of well-being at work, manifested in a lower levels of performance (Tims et al., 2015), career satisfaction (Gallagher and Hughes, 2020) and engagement (Petrou et al., 2018; Petrou et al., 2015), as well as higher levels of turnover intentions (Oprea et al., 2020) and burnout (Demerouti et al., 2015; Petrou et al., 2015).

One of the variables involved in the negative impact of this job crafting strategy can be the frustration of the three BPN. By distancing oneself from demanding job tasks, and postponing or assigning them to other colleagues, the employee lowers their chances of acquiring new skills, weakens their sense of job control, and probably alienates their work colleagues (Tims et al., 2015). In the long run, this results in an increased frustration of needs, which may lead to the deterioration of well-being at work.

### Limitations and future directions

This research is not without limitations. Although the three-wave prospective study with a few months interval design can clarify the direction between job crafting, psychological needs and well-being, it is not justified to draw valid causal inferences (Dubbelt et al., 2019). Conducting an experimental study that manipulates crafting, however, would be difficult or impossible to perform. Experimental studies are not always possible due to, among others, high complexity of research contexts or the nature of variables (e.g., the inability to manipulate variables for ethical, practical reasons, or resulting from the very nature of the constructs). A research method that allows approximate causal inferences to be drawn in the absence of the possibility of using the experimental scheme are longitudinal studies. These studies, for example, using autoregressive effects and the so-called cross-lagged effects, make it possible to examine not only the stability of effects over time, but also the approximate effects of causality while controlling variance of the remaining constructs; they also allow for tracing the direction of effects (Kline, 2016). In the past such models were even referred to as causal (Taris and Kompier, 2003). Leaving aside some criticism (Bentler, 1980; Hamaker et al., 2015), these models are currently the best possible approximation of cause-and-effect reasoning in the absence of experimental data availability. However, the psychological constructs are usually of high complexity which makes already modest effects disappear in cross-lagged models as so many other variables are controlled in the process. With this in mind and being interested in a direct approach, i.e., testing only relationships of interest, we decided to analyze our hypotheses using a strictly confirmatory approach to SEM, which is a prevailing approach to hypothesis testing in the social sciences (Collier, 2020). However, we wanted to stress that there are several ways in which the data presented could be analyzed.

In terms of generalizability, it should be noted that the results of this study were obtained from a sample of human service workers. The observed regularities relate to this group of professions only and should not be generalized to other occupational sectors, such as IT, production and transport. Another issue is the gender disproportion in the research sample. Women were over-represented, because the number of women in the health and educational sectors is significantly greater. As regards the male population, in traditionally typical male occupations, the results would be perhaps different. Age and seniority differences between the three comparison occupational groups can be also perceived as problematic. Health service workers was significantly older and more experienced than two other groups. On the other hand observed differences reflected real employment structure in Polish health sector. For example, age average in Poland, in 2021 was 53.6; nearly 52% of all nurses are aged over 51 years old, while people up to 30 years old constitute only 5.5% of the workforce. Such a high average age is largely due to the emigration of younger, well-educated Polish nurses to Western European countries and little interest in studying nursing in Poland in recent years.

A further issue is that the research was conducted during the COVID-19 pandemic; therefore, some responses may be biased by the specifics of the situation. First, during a pandemic, the organization of work and the level of professional requirements usually differ from traditional ones. For example, health-care workers struggled with a large number of atypical job stressors such as staff shortages, insufficient equipment, inadequate protection against contamination, risk of infection, social stigma, isolation, lack of contact with family, and lack of consistent information about the spread of the virus, its contagiousness and the effectiveness of its preventive measures (Fiorillo and Gorwood, 2020). These abnormal working conditions can have a significant impact on the results obtained, e.g., perceived workload and exhaustion. In the education and services branches, during the pandemic, a significant proportion of employees (at least partially) worked remotely. The possibilities of crafting their own work under these conditions also differ significantly from traditional ones.

The findings of this study point to several directions for future research. One important area involves better understanding the contextual and occupational factors that may moderate the effectiveness of different job crafting strategies. The absence of a clear link between reducing hindrance demands and exhaustion, as well as the unexpected negative association between seeking social resources and need satisfaction, suggest that job crafting may function differently depending on the nature of the job or work environment. Future studies could explore how professional context, such as the presence of inescapable job stressors or cultural norms surrounding support-seeking, influences the outcomes of job crafting behaviors. In addition, future research should consider the role of individual differences in shaping how job crafting strategies affect well-being. For example, traits such as self-efficacy, self-esteem, and sensitivity to feedback may influence whether social support is experienced as helpful or threatening. Furthermore, more detailed and process-oriented studies – including daily diary designs or qualitative interviews – could help clarify interpersonal mechanisms involved in social job crafting, such as the risk of overuse, perceptions of exploitation, or reciprocity expectations among coworkers. Finally, future work should expand the current model by including alternative mediators and examining longitudinal or reciprocal effects over time. Variables such as perceived job control, role clarity, or organizational trust may offer additional insight into how job crafting influences well-being. Researchers are also encouraged to investigate organizational-level factors, such as leadership style or team climate, that might foster or inhibit the success of crafting behaviors. Intervention studies testing job crafting training programs would be especially valuable in identifying how to promote effective and sustainable crafting strategies tailored to both individual and organizational needs.

### Practical implications

The findings of the present study yield several significant practical implications for human resource professionals and designers of workplace interventions aimed at fostering sustainable employee well-being. First, the results underscore the critical importance of promoting approach-oriented job crafting, particularly behaviors aimed at increasing structural job resources, as an effective strategy for enhancing employee engagement. This suggests that organizations should cultivate environments that empower employees to proactively shape their roles—by increasing autonomy, providing developmental opportunities, and clarifying career growth trajectories. Interventions such as job crafting workshops, participatory job design initiatives, and coaching programs may be particularly effective in equipping employees with the skills and confidence necessary to engage in meaningful crafting behaviors.

Second, the findings indicate that avoidance-oriented job crafting, especially the reduction of hindrance demands, may inadvertently undermine employee well-being by frustrating basic psychological needs. Accordingly, encouraging employees to simply eliminate stressful job elements—without addressing their underlying causes—may prove counterproductive. Instead, organizations should prioritize structural solutions to mitigate hindrance demands, such as optimizing workload distribution, fostering supportive leadership practices, and redesigning problematic tasks.

Third, the mediating role of psychological need satisfaction and frustration highlights the importance of adopting a motivational framework grounded in Self-Determination Theory. By cultivating a work environment that satisfies employees’ needs for autonomy, competence, and relatedness, organizations can not only enhance the positive effects of proactive job crafting but also buffer against the negative consequences associated with defensive crafting strategies.

Finally, these findings hold particular relevance for social service organizations, where high emotional demands and elevated burnout risks are especially prevalent. Investing in sustainable work design and proactive human resource strategies may not only improve employee well-being but also reduce turnover, preserve institutional knowledge, and ensure continuity of care for vulnerable client populations.

## Data Availability

The raw data supporting the conclusions of this article will be made available by the authors, without undue reservation.

## References

[ref1] Abu-Bader S. H. (2000). Work satisfaction, burnout, and turnover among social workers in Israel: a causal diagram. Int. J. Soc. Welf. 9, 191–200. doi: 10.1111/1468-2397.00128

[ref2] Albrecht S. L. (2015). Challenge demands, hindrance demands, and psychological need satisfaction. J. Pers. Psychol. 14, 70–79. doi: 10.1027/1866-5888/a000122, PMID: 31409215

[ref3] Baard P. P. Deci E. L. Ryan R. M. (2004). Intrinsic need satisfaction: a motivational basis of performance and well-being in two work settings. J. Appl. Soc. Psychol. 34, 2045–2068. doi: 10.1111/j.1559-1816.2004.tb02690.x

[ref4] Baka Ł. Prusik M. (2021). Towards better understanding of the harmful impact of hindrance and challenge stressors on job burnout of nurses. A one-year cross-lagged study on mediation role of work-family conflict. Front. Psychol. 12:696891. doi: 10.3389/fpsyg.2021.696891, PMID: 34603125 PMC8484705

[ref5] Baka Ł. Szulawski M. Prusik M. Kapica Ł. Najmiec A. (2022). Longitudinal relation between comprehensive job resources and three basic psychological needs at work. Int. J. Environ. Res. Public Health 19:6302. doi: 10.3390/ijerph19106302, PMID: 35627839 PMC9141893

[ref6] Bakker A. B. Demerouti E. (2017). Job demands-resources theory: taking stock and looking forward. J. Occup. Health Psychol. 22, 273–285. doi: 10.1037/ocp0000056, PMID: 27732008

[ref7] Bakker A. B. Demerouti E. Sanz-Vergel A. (2023). Job demands–resources theory: ten years later. Annu. Rev. Organ. Psychol. Organ. Behav. 10, 25–53. doi: 10.1146/annurev-orgpsych-120920-053933

[ref8] Bakker A. B. Oerlemans W. G. (2019). Daily job crafting and momentary work engagement: a self-determination and self-regulation perspective. J. Vocat. Behav. 112, 417–430. doi: 10.1016/j.jvb.2018.12.005

[ref9] Bamberger P. (2009). “Employee help-seeking: antecedents, consequences and new insights for future research” in Research in personnel and human resources management. eds. Martocchio J. J. Liao H. (Bingley: Emerald Group Publishing Limited), 49–98.

[ref10] Beehr T. A. Bowling N. A. Bennett M. M. (2010). Occupational stress and failures of social support: when helping hurts. J. Occup. Health Psychol. 15, 45–59. doi: 10.1037/a0018234, PMID: 20063958

[ref11] Bentler P. M. (1980). Multivariate analysis with latent variables: causal modeling. Annu. Rev. Psychol. 31, 419–456. doi: 10.1146/annurev.ps.31.020180.002223

[ref12] Cavanaugh M. A. Boswell W. R. Roehling M. V. Boudreau J. W. (2000). An empirical examination of self-reported work stress among U.S. managers. J. Appl. Psychol. 85, 65–74. doi: 10.1037/0021-9010.85.1.65, PMID: 10740957

[ref13] Collier J. E. (2020). Applied structural equation modeling using AMOS: Basic to advanced techniques. New York, USA: Routledge.

[ref14] Coxen L. van der Vaart L. Van den Broeck A. Rothmann S. (2021). Basic psychological needs in the work context: a systematic literature review of diary studies. Front. Psychol. 12:698526. doi: 10.3389/fpsyg.2021.698526, PMID: 34733198 PMC8558380

[ref15] Crawford E. R. LePine J. A. Rich B. L. (2010). Linking job demands and resources to employee engagement and burnout: a theoretical extension and meta-analytic test. J. Appl. Psychol. 95:834. doi: 10.1037/a0019364, PMID: 20836586

[ref16] Cropley M. Zijlstra F. R. H. (2011). “Work and rumination” in Handbook of stress in the occupations. eds. Langan-Fox J. Cooper C. L. (Cheltenham, UK: Edward Elgar Publishing), 487–501.

[ref17] De Croon E. M. Sluiter J. K. Blonk R. W. B. Broersen J. P. J. Frings-Dresen M. H. W. (2004). Stressful work, psychological job strain, and turnover: a two-year prospective cohort study of truck driver. J. Appl. Psychol. 89, 442–454. doi: 10.1037/0021-9010.89.3.442 15161404

[ref18] Deci E. L. Ryan R. M. (2000). The “what” and “why” of goal pursuits: human needs and the self-determination of behavior. Psychol. Inq. 11, 227–268. doi: 10.1207/S15327965PLI1104_01

[ref19] Deelstra J. T. Peeters M. C. W. Schaufeli W. B. Stroebe W. Zijlstra F. R. H. (2003). Receiving instrumental support at work: when help is not welcome. J. Appl. Psychol. 88, 324–331. doi: 10.1037/0021-9010.88.2.324, PMID: 12731716

[ref20] Demerouti E. Bakker A. B. Halbesleben J. R. (2015). Productive and counterproductive job crafting: a daily diary study. J. Occup. Health Psychol. 20, 457–469. doi: 10.1037/a0039002, PMID: 25798721

[ref21] Demerouti E. Bakker A. Vardakou I. Kantas A. (2003). The convergent validity of two burnout instruments: a multitrait-multimethod analysis. Eur. J. Psychol. Assess. 19, 12–23. doi: 10.1027//1015-5759.19.1.12

[ref22] Demerouti E. Xanthopoulou D. Petrou P. Karagkounis C. (2017). Does job crafting assist dealing with organizational changes due to austerity measures? Two studies among Greek employees. Eur J Work Organ Psychol 26, 574–589. doi: 10.1080/1359432X.2017.1325875

[ref23] Dubbelt L. Demerouti E. Rispens S. (2019). The value of job crafting for work engagement, task performance, and career satisfaction: longitudinal and quasi-experimental evidence. Eur. J. Work Organ. Psychol. 28:632. doi: 10.1080/1359432X.2019.1576632

[ref24] Edwards J. R. Van Harrison R. (1993). Job demands and worker health: three-dimensional reexamination of the relationship between person-environment fit and strain. J. Appl. Psychol. 78, 628–648. doi: 10.1037/0021-9010.78.4.628, PMID: 8407706

[ref25] Fiorillo A. Gorwood P. (2020). The consequences of the COVID-19 pandemic on mental health and implications for clinical practice. Eur. Psychiatry 63:e32. doi: 10.1192/j.eurpsy.2020.35, PMID: 32234102 PMC7156565

[ref26] Frederick D. E. VanderWeele T. J. (2020). Longitudinal meta-analysis of job crafting shows positive association with work engagement. Cogent Psychol. 7:1746733. doi: 10.1080/23311908.2020.1746733

[ref27] Gallagher C. M. Hughes I. M. (2020). Bearing the burden: outcomes and moderators of social burden in the workplace. Occup. Health Sci. 4, 123–138. doi: 10.1007/s41542-020-00063-4

[ref28] Gaskin J. J. James M. Lim J. (2020). Indirect effects, Amos plugin. Gaskination’s StatWiki.

[ref29] Ghahramani S. Lankarani K. B. Yousefi M. Heydari K. Shahabi S. Azmand S. (2021). A systematic review and meta-analysis of burnout among healthcare workers during COVID-19. Front. Psych. 12:758849. doi: 10.3389/fpsyt.2021.758849, PMID: 34858231 PMC8631719

[ref30] Gillet N. Fouquereau E. Forest J. Brunault P. Colombat P. (2012). The impact of organizational factors on psychological needs and their relations with well-being. J. Bus. Psychol. 27, 437–450. doi: 10.1007/s10869-011-9253-2

[ref31] Giménez-Bertomeu V. M. Caravaca-Sánchez F. de Alfonseti-Hartmann N. Ricoy-Cano A. J. (2024). Burnout among social workers in social services: a systematic review and meta-analysis of prevalence. J. Soc. Serv. Res. 50, 664–683. doi: 10.1080/01488376.2024.2371847

[ref32] Gray C. E. Spector P. E. Lacey K. N. Young B. G. Jacobsen T. Taylor M. R. (2020). Helping may be harming: unintended negative consequences of providing social support. Work Stress. 34, 359–385. doi: 10.1080/02678373.2019.1695294, PMID: 38075181 PMC10701713

[ref33] Hamaker E. L. Kuiper R. M. Grasman R. P. (2015). A critique of the cross-lagged panel model. Psychol. Methods 20, 102–116. doi: 10.1037/a0038889, PMID: 25822208

[ref34] Hayes A. F. (2018). Introduction to mediation, moderation, and conditional process analysis. A regression-based approach. New York, NY, USA: Guilford.

[ref35] Hobfoll S. E. Halbesleben J. Neveu J. P. Westman M. (2018). Conservation of resources in the organizational context: the reality of resources and their consequences. Annu. Rev. Organ. Psychol. Organ. Behav. 5, 103–128. doi: 10.1146/annurev-orgpsych-032117-104640

[ref36] Hom P. W. Lee T. Shaw J. D. Hausknecht J. P. (2017). One hundred years of employee turnover theory and research. J. Appl. Psychol. 102, 530–545. doi: 10.1037/apl0000103, PMID: 28125259

[ref37] Kim H. Stoner M. (2008). Burnout and turnover intention among social workers: effects of role stress, job autonomy and social support. Adm. Soc. Work. 32, 5–25. doi: 10.1080/03643100801922357

[ref38] Kline R. B. (2016). Principles and practice of structural equation modeling. 4th Edn. New York, NY, USA: Guilford Press.

[ref39] Lichtenthaler P. W. Fischbach A. (2019). A meta-analysis on promotion- and prevention-focused job crafting. Eur. J. Work Organ. Psychol. 28, 30–50. doi: 10.1080/1359432X.2018.1527767

[ref40] Little T. D. (2024). Longitudinal structural equation modeling. 2nd Edn. New York, USA: The Guilford Press.

[ref41] Maslach C. Schaufeli W. B. Leiter M. P. (2001). Job burnout. Annu. Rev. Psychol. 52, 397–422. doi: 10.1146/annurev.psych.52.1.397, PMID: 11148311

[ref42] Mathieu M. Eschleman K. J. Cheng D. (2019). Meta-analytic and multiwave comparison of emotional support and instrumental support in the workplace. J. Occup. Health Psychol. 24, 387–409. doi: 10.1037/ocp0000135, PMID: 30335420

[ref43] Maxwell S. E. Cole D. A. Mitchell M. A. (2011). Bias in cross-sectional analyses of longitudinal mediation: partial and complete mediation under an autoregressive model. Multivar. Behav. Res. 46, 816–841. doi: 10.1080/00273171.2011.606716, PMID: 26736047

[ref44] Mercado M. Wachter K. Schuster R. C. Mathis C. M. Johnson E. Davis O. I. (2022). A cross-sectional analysis of factors associated with stress, burnout and turnover intention among healthcare workers during the COVID-19 pandemic in the United States. Health Soc. Care Community 30, 2690–2701. doi: 10.1111/hsc.13712 35037346

[ref45] O’Connor K. Muller Neff D. Pitman S. (2018). Burnout in mental health professionals: a systematic review and meta-analysis of prevalence and determinants. Eur. Psychiatry 53, 74–99. doi: 10.1016/j.eurpsy.2018.06.003, PMID: 29957371

[ref46] Olafsen A. H. Frølund C. W. (2018). Challenge accepted! Distinguishing between challenge and hindrance demands. J. Manag. Psychol. 33, 345–357. doi: 10.1108/JMP-04-2017-0143

[ref47] Oprea B. Păduraru L. Iliescu D. (2020). Job crafting and intent to leave: the mediating role of meaningful work and engagement. J. Career Dev. 49:18666. doi: 10.1177/0894845320918666

[ref48] Ozamiz-Etxebarria N. Legorburu Fernandez I. Lipnicki D. M. Idoiaga Mondragon N. Santabárbara J. (2023). Prevalence of burnout among teachers during the COVID-19 pandemic: a meta-analysis. Int. J. Environ. Res. Public Health 20, 1–13. doi: 10.3390/ijerph20064866 PMC10049404 36981775

[ref49] Peeters M. C. W. Buunk B. P. Schaufeli W. B. (1995). Social interactions and feelings of inferiority among correctional officers: a daily event-recording approach. J. Appl. Soc. Psychol. 25, 1073–1089.

[ref50] Petrou P. Demerouti E. Schaufeli W. B. (2015). Job crafting in changing organizations: antecedents and implications for exhaustion and performance. J. Occup. Health Psychol. 20, 470–480. doi: 10.1037/a0039003, PMID: 25798717

[ref51] Petrou P. Demerouti E. Schaufeli W. B. (2018). Crafting the change: the role of employee job crafting behaviors for successful organizational change. J. Manag. 44, 1766–1792. doi: 10.1177/0149206315624961

[ref52] Pharris A. B. Munoz R. T. Hellman C. M. (2022). Hope and resilience as protective factors linked to lower burnout among child welfare workers. Child Youth Serv. Rev. 136:106424. doi: 10.1016/j.childyouth.2022.106424

[ref53] Podsakoff N. P. LePine J. A. LePine M. A. (2007). Differential challenge stressor-hindrance stressor relationships with job attitudes, turnover intentions, turnover, and withdrawal behavior: a meta-analysis. J. Appl. Psychol. 92:438. doi: 10.1037/0021-9010.92.2.438, PMID: 17371090

[ref54] Ravalier J. M. McFadden P. Boichat C. Clabburn O. Moriarty J. (2021). Social worker well-being: a large mixed-methods study. Br. J. Soc. Work. 51, 297–317. doi: 10.1093/bjsw/bcaa078

[ref55] Roczniewska M. A. Puchalska-Kamińska M. (2017). Are managers also crafting leaders? The link between organizational rank, autonomy, and job crafting. Pol. Psychol. Bull. 48, 198–211. doi: 10.1515/ppb-2017-0023

[ref56] Ryan R. M. Deci E. L. (2000). Self-determination theory and the facilitation of intrinsic motivation, social development, and well-being. Am. Psychol. 55, 68–78. doi: 10.1037/0003-066X.55.1.68, PMID: 11392867

[ref57] Ryan R. M. Deci E. L. (2002) Overview of self-determination theory: an organismic dialectical perspective. In: Deci E. L. Ryan R. M., editors. Handbook of self-determination research. New York, NY: University of Rochester Press. pp. 3–33.

[ref58] Ryan R. M. Deci E. L. (2017). Self-determination theory: Basic psychological needs in motivation, development, and wellness. New York, NY, USA: The Guilford Press.

[ref59] Sá M. M. Azevedo R. (2020). “Social workers’ exposure to psychosocial risks—a case study” in Occupational and environmental safety and health II. eds. Arezes P.. (Cham: Springer), 605–613.

[ref60] Schaufeli W. B. Salanova M. González-Romá V. Bakker A. B. (2002). The measurement of engagement and burnout: a two sample confirmative factor analytic approach. J. Happiness Stud. 3, 71–92. doi: 10.1023/A:1015630930326

[ref61] Schultz P. P. Ryan R. M. Niemiec C. P. Legate N. Williams G. C. (2015). Mindfulness, work climate, and psychological need satisfaction in employee well-being. Mindfulness 6, 971–985. doi: 10.1007/s12671-014-0338-7

[ref62] Schumacker R. E. Lomax R. G. (2004). A beginner’s guide to structural equation modeling. 2nd Edn. Mahwah, NJ: Erlbaum.

[ref63] Sharma S. (1996). Applied multivariate techniques. New York, USA: John Wiley and Sons Inc.

[ref64] Shen X. Xu H. Feng J. Ye J. Lu Z. Gan Y. (2022). The global prevalence of burnout among general practitioners: a systematic review and meta-analysis. Fam. Pract. 39, 943–950. doi: 10.1093/fampra/cmab180, PMID: 35089320

[ref65] Skaalvik E. M. Skaalvik S. (2007). Dimensions of teacher self-efficacy and relations with strain factors, perceived collective teacher efficacy, and teacher burnout. J. Educ. Psychol. 99, 611–625. doi: 10.1037/0022-0663.99.3.611

[ref66] Slemp G. R. Vella-Brodrick D. A. (2014). Optimising employee mental health: the relationship between intrinsic need satisfaction, job crafting, and employee well-being. J. Happiness Stud. 15, 957–977. doi: 10.1007/s10902-013-9458-3

[ref67] Straussner S. L. A. Senreich E. Steen J. T. (2018). Wounded healers: a multistate study of licensed social workers’ behavioral health problems. Soc. Work 63, 125–133. doi: 10.1093/sw/swy012, PMID: 29425335 PMC6042294

[ref68] Taris T. W. Kompier M. (2003). Challenges in longitudinal designs in occupational health psychology. Scand. J. Work Environ. Health 29, 1–4. doi: 10.5271/sjweh.697, PMID: 12630429

[ref69] Tims M. Bakker A. B. (2010). Job crafting: towards a new model of individual job redesign. S. Afr. J. Ind. Psychol. 36, 1–9. doi: 10.4102/sajip.v36i2.841

[ref70] Tims M. Bakker A. B. Derks D. (2012). Development and validation of the job crafting scale. J. Vocat. Behav. 80, 173–186. doi: 10.1016/j.jvb.2011.05.009

[ref71] Tims M. Bakker A. B. Derks D. (2013). The impact of job crafting on job demands, job resources, and well-being. J. Occup. Health Psychol. 18, 230–240. doi: 10.1037/a0032141, PMID: 23506549

[ref72] Tims M. Bakker A. B. Derks D. (2015). Job crafting and job performance: a longitudinal study. Eur. J. Work Organ. Psychol. 24, 914–928. doi: 10.1080/1359432X.2014.969245

[ref73] Toyama H. Upadyaya K. Salmela-Aro K. (2022). Job crafting and well-being among school principals: the role of basic psychological need satisfaction and frustration. Eur. Manag. J. 40:3. doi: 10.1016/j.emj.2021.10.003

[ref74] Travaglianti F. Babic A. Hansez I. (2016). The role of work-related needs in the relationship between job crafting, burnout and work engagement. S. Afr. J. Ind. Psychol. 42:1308. doi: 10.4102/sajip.v42i1.1308

[ref75] Trépanier S. G. Forest J. Fernet C. Austin S. (2015). On the psychological and motivational processes linking job characteristics to employee functioning: insights from self-determination theory. Work Stress. 29, 286–305. doi: 10.1080/02678373.2015.1074957

[ref76] Van den Broeck A. Ferris D. L. Chang C. H. Rosen C. C. (2016). A review of self-determination theory’s basic psychological needs at work. J. Manag. 42, 1195–1229. doi: 10.1177/0149206316632058, PMID: 40406039

[ref77] Van den Broeck A. Vansteenkiste M. De Witte H. Lens W. (2008). Explaining the relationships between job characteristics, burnout, and engagement: the role of basic psychological need satisfaction. Work Stress. 22, 277–294. doi: 10.1080/02678370802393672

[ref78] Van Hooff M. L. M. De Pater I. E. (2019). Daily associations between basic psychological need satisfaction and well-being at work: the moderating role of need strength. J. Occup. Organ. Psychol. 92, 1027–1035. doi: 10.1111/joop.12260

[ref79] Vander Elst T. Broeck A. De Witte H. De Cuyper N. (2012). The mediating role of frustration of psychological needs in the relationship between job insecurity and work-related well-being. Work Stress. 26, 252–271. doi: 10.1080/02678373.2012.703900

[ref80] Vansteenkiste M. Ryan R. M. (2013). On psychological growth and vulnerability: basic psychological need satisfaction and need frustration as a unifying principle. J. Psychother. Integr. 23, 263–280. doi: 10.1037/a0032359

[ref81] Warburton V. E. Wang J. C. K. Bartholomew K. J. Tuff R. L. Bishop K. C. M. (2020). Need satisfaction and need frustration as distinct and potentially co-occurring constructs: need profiles examined in physical education and sport. Motiv. Emot. 44, 54–66. doi: 10.1007/s11031-019-09798-2

[ref82] Williams K. D. (2009). Ostracism: A temporal need-threat model. Adv Exp Soc Psychol 41, 275–314. doi: 10.1016/S0065-2601(08)00406-1

[ref83] Woo T. Ho R. Tang A. Tam W. (2020). Global prevalence of burnout symptoms among nurses: a systematic review and meta-analysis. J. Psychiatr. Res. 123, 9–20. doi: 10.1016/j.jpsychires.2019.12.015, PMID: 32007680

[ref84] Wrzesniewski A. Dutton J. E. (2001). Crafting a job: revisioning employees as active crafters of their work. Acad. Manag. Rev. 26, 179–201. doi: 10.2307/259118

[ref85] Zhang F. Parker S. K. (2019). Reorienting job crafting research: a hierarchical structure of job crafting concepts and integrative review. J. Organ. Behav. 40, 126–146. doi: 10.1002/job.2332

[ref86] Zhao X. Lynch J. G. Jr. Chen Q. (2010). Reconsidering baron and Kenny: myths and truths about mediation analysis. J. Consum. Res. 37, 197–206. doi: 10.1086/651257

