# Supplementary material for: Insight into development of job-related well-being: the role of four job crafting strategies and psychological needs
Source: Front Psychol. 2025 Jun 26;16:1487043. doi: 10.3389/fpsyg.2025.1487043 (PMC12241067; doi:10.3389/fpsyg.2025.1487043)
Supplement: Supplementary file 1 [file Supplementary_file_1.docx]

Appendix A

Table 1A

*Zero-order Correlations for Study Variables Across Different Times of Measurement,* N *= 839*

| **Construct** | **(1)** | **(2)** | **(3)** | **(4)** | **(5)** | **(6)** | **(7)** | **(8)** |
| --- | --- | --- | --- | --- | --- | --- | --- | --- |
| **Time 2** | | | | | | | | |
| **Time 1** |  |  |  |  |  |  |  |  |
| 1. Structural job resources | .28*** | .08* | .22*** | .03 | .15*** | -.07* | .22*** | -.13*** |
| 2. Social job resources | .15*** | .37*** | .30*** | .08* | -.10** | .14*** | .15*** | -.02 |
| 3. Challange demands | .25*** | .26*** | .34*** | .10** | .05 | .08* | .22*** | -.09* |
| 4. Hindrance demands | .05 | .13*** | .15*** | .26*** | .03 | .07* | .06 | .01 |
| 5. Needs satisfaction | .13*** | -.06 | .04 | .01 | .26*** | -.17*** | .18*** | -.17*** |
| 6. Needs frustration | -.06 | .14*** | .08* | .08* | -.17*** | .25*** | .09*** | .12*** |
| 7. Work engagement | .27*** | .09** | .22*** | .06 | .20*** | -.07* | .35*** | -.14*** |
| 8. Exhaustion | -.16*** | .03 | -.06 | -.03 | -.14*** | .15*** | -.03 | .29*** |
| **Time 3** | | | | | | | | |
| **Time 1** |  |  |  |  |  |  |  |  |
| 1. Structural job resources | .28*** | .11** | .22*** | .00 | .12** | -.04 | .22*** | -.09** |
| 2. Social job resources | .13*** | .35*** | .24*** | .08* | -.07* | .09* | .07* | .04 |
| 3. Challange demands | .23*** | .27*** | .32*** | .08* | .04 | .06 | .18*** | -.03 |
| 4. Hindrance demands | -.01 | .08* | .07* | .19*** | .06 | .03 | .16*** | -.01 |
| 5. Needs satisfaction | .13*** | .00 | .09* | .10** | .21*** | -.13*** | .24*** | -.13*** |
| 6. Needs frustration | -.04 | .10** | .07* | .04 | -.14*** | .22*** | .01 | .18*** |
| 7. Work engagement | .23*** | .15*** | .20*** | .10** | .13*** | -.03 | .28*** | -.11** |
| 8. Exhaustion | -.07* | .04 | .00 | .00 | -.05 | .08* | -.07 | .20*** |
| **Time 3** | | | | | | | | |
|  | | | | | | | | |
| **Time 2** |  |  |  |  |  |  |  |  |
| 1. Structural job resources | .33*** | .16*** | .27*** | .00 | .20*** | -.01 | .26*** | -.11** |
| 2. Social job resources | .13*** | .30*** | .22*** | .06 | -.01 | .08* | .09** | .00 |
| 3. Challange demands | .23*** | .29*** | .37*** | .06 | .08* | .09** | .19*** | .01 |
| 4. Hindrance demands | .01 | .09* | .09* | .26*** | .12** | -.03 | .16*** | -.03 |
| 5. Needs satisfaction | .16*** | -.08* | .06 | .14*** | .35*** | -.20*** | .26*** | -.21*** |
| 6. Needs frustration | -.07 | .12*** | .10*** | -.02 | -.25*** | .31*** | -.11** | .26*** |
| 7. Work engagement | .29*** | .23*** | .26*** | .06 | .23*** | .00 | .40*** | -.13*** |
|  |  |  |  |  |  |  |  |  |
|  |  |  |  |  |  |  |  |  |
